# Sodium channels in the Cx43 gap junction perinexus may constitute a cardiac ephapse: an experimental and modeling study

**DOI:** 10.1007/s00424-014-1675-z

**Published:** 2015-01-13

**Authors:** Rengasayee Veeraraghavan, Joyce Lin, Gregory S. Hoeker, James P. Keener, Robert G. Gourdie, Steven Poelzing

**Affiliations:** 1 Virginia Tech Carilion Research Institute, and Center for Heart and Regenerative Medicine, Virginia Polytechnic University, Roanoke, VA 24016 USA; 2 School of Biomedical Engineering and Sciences, Virginia Polytechnic University, Blacksburg, VA USA; 3 Department of Mathematics, California Polytechnic State University, San Luis Obispo, CA USA; 4 Department of Mathematics, University of Utah, 155 South 1400 East, Salt Lake City, UT 84112 USA

**Keywords:** Cardiac conduction, Arrhythmia, Sodium channels, Gap junctions, Ephaptic coupling

## Abstract

It has long been held that electrical excitation spreads from cell-to-cell in the heart via low resistance gap junctions (GJ). However, it has also been proposed that myocytes could interact by non-GJ-mediated “ephaptic” mechanisms, facilitating propagation of action potentials in tandem with direct GJ-mediated coupling. We sought evidence that such mechanisms contribute to cardiac conduction. Using super-resolution microscopy, we demonstrate that Na_v_1.5 is localized within 200 nm of the GJ plaque (a region termed the perinexus). Electron microscopy revealed close apposition of adjacent cell membranes within perinexi suggesting that perinexal sodium channels could function as an ephapse, enabling ephaptic cell-to-cell transfer of electrical excitation. Acute interstitial edema (AIE) increased intermembrane distance at the perinexus and was associated with preferential transverse conduction slowing and increased spontaneous arrhythmia incidence. Inhibiting sodium channels with 0.5 μM flecainide uniformly slowed conduction, but sodium channel inhibition during AIE slowed conduction anisotropically and increased arrhythmia incidence more than AIE alone. Sodium channel inhibition during GJ uncoupling with 25 μM carbenoxolone slowed conduction anisotropically and was also highly proarrhythmic. A computational model of discretized extracellular microdomains (including ephaptic coupling) revealed that conduction trends associated with altered perinexal width, sodium channel conductance, and GJ coupling can be predicted when sodium channel density in the intercalated disk is relatively high. We provide evidence that cardiac conduction depends on a mathematically predicted ephaptic mode of coupling as well as GJ coupling. These data suggest opportunities for novel anti-arrhythmic therapies targeting noncanonical conduction pathways in the heart.

**Electronic supplementary material:**

The online version of this article (doi:10.1007/s00424-014-1675-z) contains supplementary material, which is available to authorized users.

## Introduction

Cardiac conduction is the process by which electrical impulses flow through the heart, triggering synchronous contraction. For over 50 years, cardiac electrical coupling has been thought to be exclusively electrotonic, with gap junction (GJ) channels envisaged as providing low resistance electrical pathways between myocytes [7]. While there is much evidence correlating the loss of GJs with slowed conduction in cardiac disease, the relationship between GJ coupling and conduction velocity (CV) is controversial [6, 13, 25, 39, 40].

Theoretical models have raised the possibility of myocytes being coupled ephaptically, without recourse to GJ intercellular communication [21, 24, 35], as occurs in other tissues, such as neurons [2], the retina [43], and uterine myometrium [45]. Ephaptic coupling involves cell-to-cell transfer of electrical activation via transient extracellular electric fields or ion accumulation/depletion within a confined interstitial space between two closely apposed cells [19, 21, 24, 35, 37]. However, the ephaptic hypothesis in heart has lacked experimental support, as well as an identifiable functional unit that could mediate this noncanonical mechanism for cell-to-cell transfer of action potential, i.e., an ephapse.

In silico studies have suggested that ephaptic coupling is possible in the myocardium if the membranes of adjacent myocytes are closely apposed (≈10 nm) and if voltage-gated sodium channels are present at sufficient density [18, 19, 24]. The purposes of this manuscript are to 1) identify a sub-domain within the intercalated disk (ID) which possesses these characteristics, 2) determine if these sub-domains are subject to dynamic regulation, 3) demonstrate novel conduction behavior that cannot be fully explained by GJ coupling, and 4) adapt mathematical models of cardiac conduction to probe whether theoretical ephaptic mechanisms are compatible with our experimental data.

## Methods

The investigation conforms to the *Guide for the Care and Use of Laboratory Animals* published by the US National Institutes of Health (NIH Publication No. 85–23, revised 1996). All animal study protocols were approved by Institutional Animal Care and Use Committee (IACUC) at the Virginia Polytechnic University.

### Animal preparations

Adult male guinea pigs (800–1000 g) were anesthetized [30 mg/kg sodium pentobarbital (Nembutal) IP], their hearts extracted, ventricles isolated and frozen for cryosectioning, or perfused (at 40–55 mmHg) as Langendorff preparations with oxygenated Tyrode’s solution (containing, in mM, CaCl_2_ 1.25, NaCl 140, KCl 4.5, dextrose 5.5, MgCl_2_ 0.7, HEPES 10; pH 7.41) at 37 °C as previously described [27, 41]. In all optical mapping experiments, control Tyrode’s solution was perfused for 35 min.

Acute interstitial edema (AIE) was induced by perfusion of mannitol (26.1 g/l/143.2 mOsm) while GJ and the sodium current (I_Na_) were respectively inhibited by carbenoxolone (CBX; 25 μM) and flecainide (Flec; 0.5 μM). Time control experiments were perfused for 30 min with either mannitol, CBX, or Flec (*n* = 3 hearts/intervention). For experiments with more than one intervention, the first measurement was made at 10 min, then the measurement with the dual intervention was made after an additional 10 min. Preparations were paced from the anterior left ventricular (LV) epicardium (midapicobasal) at a basic cycle length of 300 ms with 1 ms pulses at 1.5 times the pacing threshold as described previously [41].

### Transmission electron microscopy

Sections were cut from 5 mm cubes of tissue taken from the anterior LV free wall of adult guinea pig hearts and fixed overnight in 2 % gluteraldehyde. Transmission electron micrographs (TEM) of the ID, particularly GJ and perinexal regions were obtained at ×150,000 magnification on a JEOL 200CX electron microscope. Intermembrane distance at perinexal and non-perinexal sites was quantified using ImageJ (NIH, http://rsbweb.nih.gov/ij/).

For immunoelectron microscopy, fixed ventricular samples were embedded in lowicryl, ultrathin sectioned, and labeled using a rabbit polyclonal antibody directed against connexin43 (Cx43; Sigma) and anti-rabbit antibodies second-labeled with immunogold particles as we have previously described [3].

### Fluorescent immunolabeling

Tissue was fixed in methanol at −20 °C for 5 min, cryosectioned (5 μm sections), and immunofluorescent staining was performed as previously described [28, 29]. Samples were labeled with mouse anti-Cx43 (Millipore MAB3067, 1:100) and rabbit polyclonal antibody against the cardiac voltage-gated sodium channel (Na_v_1.5; kindly provided by Dr. Peter Mohler, 1:100). For confocal microscopy, samples were then labeled with goat anti-mouse AlexaFluor 546 (1:4000) and goat anti-rabbit AlexaFluor 488 (1:4000) secondary antibodies. For super-resolution microscopy, samples were labeled with goat anti-rabbit Chromeo 505 (1:100) and anti-mouse biotin (1:200) followed by streptavidin-conjugated Horizon V500 (1:100) secondary antibodies.

### Confocal/gSTED microscopy and image analysis

Immunolabeled samples were imaged using a TCS SP8 laser scanning confocal microscope equipped with gated Stimulated emission depletion (gSTED) modules, a Plan Apochromat 63×/1.4 numerical aperture oil immersion objective, Leica HyD hybrid detectors and a 592-nm STED depletion laser (Leica, Buffalo Grove, IL). Individual fluorophores were imaged sequentially with the excitation wavelength switching at the end of each frame. Likewise, two-color gSTED super-resolution and confocal images were obtained sequentially as z-stacks (with a step size of 10 nm) and processed using Huygens STED deconvolution software (Scientific Volume Imaging, Hilversum, The Netherlands). This enabled a maximum lateral full width at half maximum resolution of 22 nm using gSTED [5, 32]. Three micrographs were obtained from each tissue section from three hearts for a total of nine images with myocytes in longitudinal orientation along the plane of the section.

Images were analyzed using custom software written in Matlab (MathWorks, Natick, MA). Briefly, images were thresholded by Otsu’s method and clusters identified using a nearest-neighbor algorithm (Matlab function: bwconncomp). Geometric properties including centroid position and area were calculated for clusters thus identified (Matlab function: regionprops). For each Cx43 cluster, a list of Na_v_1.5 clusters was compiled which included those which overlapped directly and those which lay within the perinexal region (defined as extending 200 nm from the edge of the Cx43 signal). The 200 nm width of the perinexus used here was based on data obtained from previously published immunofluorescence as well as in situ triton extraction [29]. Additionally, such use of immunofluorescence to identify the edge of GJ has been previously validated by comparison with electron microscopy [11].

### Electrocardiography

A volume-conducted bath ECG was obtained using a silver chloride anode located ∼2 cm from the right ventricular (RV) lateral wall, a cathode located ∼2 cm from the lateral wall of the LV, and a common ground at the back of the bath. ECGs were recorded at 1 kHz. Incidence of spontaneous ventricular tachycardias (VTs) was quantified. All VTs observed persisted for at least 1 min.

### Optical mapping

Conduction velocity (CV) and anisotropic ratio (AR; the ratio of longitudinal to transverse CV) were quantified by optical voltage mapping using the voltage sensitive dye di-4-ANEPPS (15 μM) as previously described [27, 41]. Briefly, the preparation was stained by direct coronary perfusion for 10 min, then excited by three 60-LED light sources (RL5-A9018, Superbrightleds, St. Louis, MO) fitted with 510 ± 5 nm bandpass filters (Chroma, Rockingham, VT). Fluoresced light was filtered using a 610-nm longpass filter (Newport, Irvine, CA) before being recorded with a SciMedia MiCam02 HS CCD camera (SciMedia, Irvine CA) in a tandem lens configuration capable of resolving membrane potential changes as small as 2 mV from 90 × 60 sites (16.5 × 12 mm) simultaneously at 1 kHz temporal resolution.

Motion was reduced by perfusion of 7.5 mM 2,3-butanedione monoxime (BDM). The anterior epicardium was mechanically stabilized against the front wall of the perfusion chamber. Activation time was defined as the time of the maximum first derivative of the action potential as described previously [10].

### Mathematical modeling

Conduction was simulated in a sheet of 3-dimensional myocytes using a previously published model [20]. The microdomain formulation separates myocytes with GJ and includes inhomogeneities in the extracellular spacing and ionic channel distribution around the cellular membrane. Briefly, cardiac myocytes were represented as rectangular prisms with corner inclusions and were organized into a sheet to simulate anisotropic propagation. Cells were coupled via GJ located at the ends of myocytes.

The discretized equations for the extracellular space followed current conservation, balancing transmembrane currents with current due to extracellular potential gradients. For the intracellular space, each cell with domain Ω and boundary ∂Ω followed the governing equations:

$$ {\nabla}^2{\phi}_i=0\ \mathrm{i}\mathrm{n}\ \Omega $$

$$ {\sigma}_i\nabla {\phi}_i\bullet \widehat{n} = -{C}_m\frac{\partial \left({\phi}_i-{\phi}_e\right)}{\partial t}-{I}_{ion}\left({\phi}_i-{\phi}_e,\ x\right)+g(x)\left({\phi}_n-{\phi}_i\right)\ \mathrm{on}\ \partial \Omega $$

where ${\phi}_i$ and ${\phi}_e$ represent the intracellular and extracellular potentials, respectively, ${\upsigma}_i$ is the intracellular conductivity, ${C}_m$ is the membrane capacitance, ${I}_{ion}$ represents the transmembrane ionic currents, $g(x)$ is the GJ coupling with any neighbors ${\phi}_n$, and $\widehat{n}$ is the unit outward normal on the cell membrane.

To numerically discretize the intracellular space, we assumed the cell is isopotential in the direction orthogonal to the plane of the sheet, and a node was placed in each corner of each cell. Using a finite element discretization with linearly interpolating triangular elements, we employed the Crank-Nicolson scheme in time and cell-centered finite differences for the spatial derivatives in the extracellular space. Parameter values for the experimental conditions are summarized in Table 1.

**Table 1:** Microdomain model parameters

Structure		
Cell length		101 μm
Cell width		24.1 μm
Cellular offset		50 % transverse, 20 % longitudinal
Junctional sodium current density		11 to 90 % of total
Nominal conductances		
GJ coupling		${\overline{g}}_{\mathrm{j}}$ = 100 mS/cm^2^
Extracellular junctional width		15 nm
Extracellular lateral width		0.1 μm
Lateral extracellular conductivity (${\overline{\sigma}}_{\mathrm{eff}e}$)		159.1 mS/cm
Junctional extracellular conductivity (${\overline{\sigma}}_{\mathrm{effj}}$)		17.8 mS/cm
Interventions		
I_Na_ inhibition	Sodium channel conductance (g_Na_)	86 % nominal
GJ uncoupling	${\overline{g}}_{\mathrm{j}}$	50 mS/cm^2^ (50 % nominal)
AIE	${\overline{\sigma}}_{\mathrm{eff}e}$	203.5 mS/cm
	${\overline{\sigma}}_{\mathrm{effj}}$	62.8 mS/cm

Current was injected in the direction of interest (either transverse or longitudinal). Once an action potential was initiated, no flux boundary conditions were imposed. The propagation speed was calculated by tracking the action potential wave front. The following parameters were tuned to match experimental CV and AR values for control and AIE conditions: lateral and junctional extracellular conductances (${\overline{\sigma}}_{\mathrm{effe}}$ and ${\overline{\sigma}}_{\mathrm{effj}}$), GJ coupling (${\overline{g}}_{\mathrm{j}}$), cell size, sodium and potassium peak conductances (g_Na_ and g_K_), and cellular distribution of sodium channels. Intercellular coupling was modeled only at end-to-end contacts between myocytes based on previous reports [14]. The model was used to predict the effects on conduction and conduction anisotropy secondary to individually modulating GJ, AIE, and I_Na_, and simultaneously modulating combinations of GJ, AIE, and I_Na_. For a detailed account of the validation and characterization of our microdomain model, the reader is referred to our previous article [20].

### Statistical analysis

Statistical analysis of the data was performed using a two-tailed Student’s *t* test for paired and unpaired data or a single factor ANOVA. The Šidák correction was applied to adjust for multiple comparisons. Fisher’s exact test was used to test differences in nominal data. A *p* < 0.05 was considered statistically significant. All values are reported as mean ± standard error unless otherwise noted.

## Results

### ID ultrastructure

**Fig. 1:**
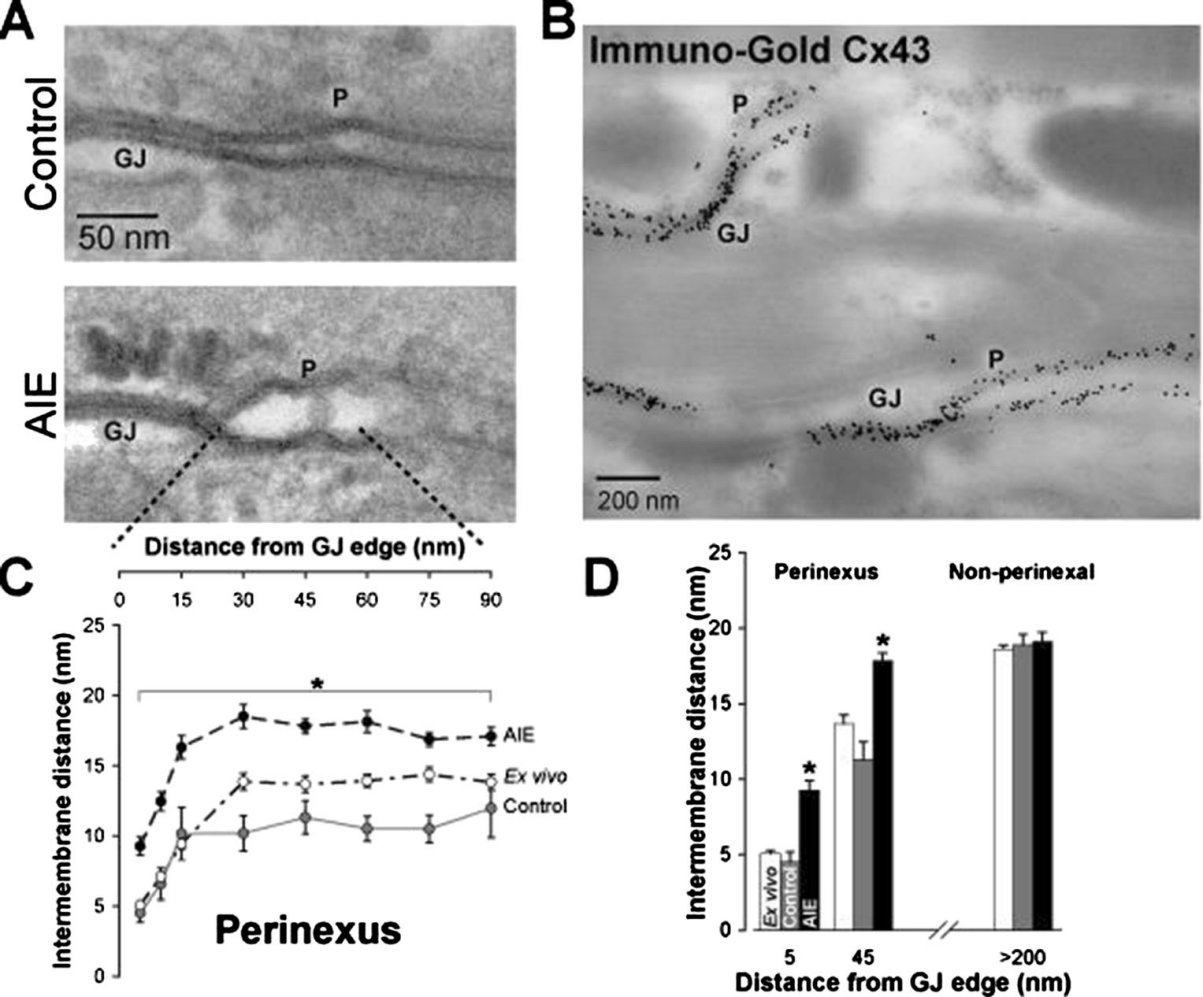
The perinexus extends up to 200 nm from the GJ edge, can be modulated, and is rich in Cx43. **a** Representative TEM of ID regions showing GJ and perinexus (*P*) from control and AIE ventricles. **b** Cx43 Immuno-EM demonstrates Cx43 labeling in junctional (*GJ*) and non-junctional membrane of the perinexus. **c** Intermembrane distance vs. distance from GJ edge within the perinexus (*n* = 3 hearts/group, *p < 0.05 vs. control at all distances—determined by ANOVA). **d** AIE increased intermembrane distance at perinexal, but not at non-perinexal sites within the ID (*p < 0.05 vs. control)

The perinexus is an ID microdomain extending ∼200 nm from the GJ plaque edge where non-junctional Cx43 connexons were previously identified [28]. Representative TEM micrographs show the membranes of adjacent cells separating at the GJ edge (Fig. 1a). Immunogold Cx43 labeling demonstrates labeling within GJ with less dense labeling extending into the non-junctional membranes comprising the perinexus (Fig. 1b). Under control conditions, the intermembrane distance at sites 5–90 nm from the GJ plaque edge consistently averaged 10 nm or less (Fig. 1c), and similar values were obtained from freshly isolated ventricles (ex vivo). Overall, there were no significant differences in membrane separation between ex vivo and control conditions at 5, 45, and greater than 200 nm from the GJ plaque (Fig. 1d). We then repeated these measurements during mannitol-induced AIE, a condition we previously demonstrated to increase gross interstitial volume and anisotropically slow conduction [42]. AIE increased perinexal membrane separation at 5 and 45 nm but not at distances along ID membranes further than 200 nm from the GJ plaque edge (Fig. 1a, c).

### Sodium channel localization

**Fig. 2:**
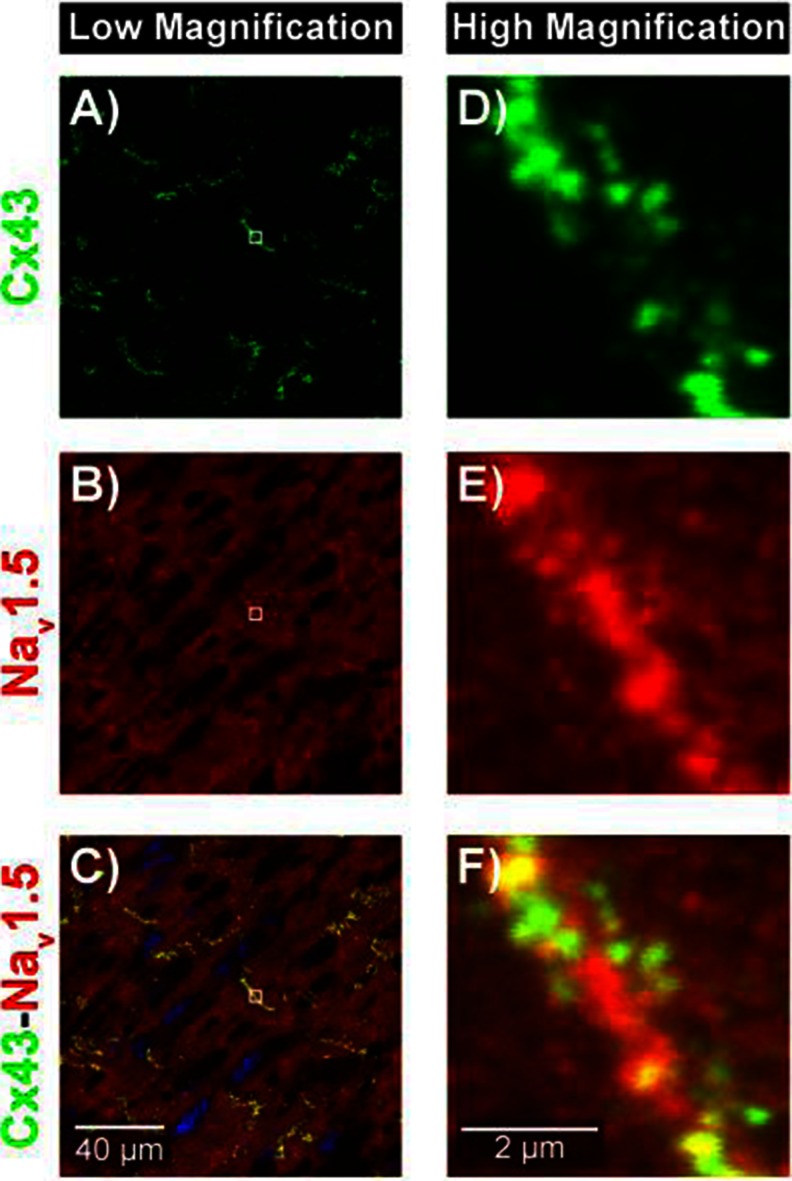
Na_v_1.5 at the ID. Representative confocal images of **a** Cx43, **b** Na_v_1.5, and **c** overlay demonstrate co-localization of Cx43 and Na_v_1.5 in ventricular sections. **d**–**f** High magnification views of regions highlighted in **a–c** by *white boxes* indicate Cx43, and Na_v_1.5 are localized in IDs but have distinct subcellular compartmentation

**Fig. 3:**
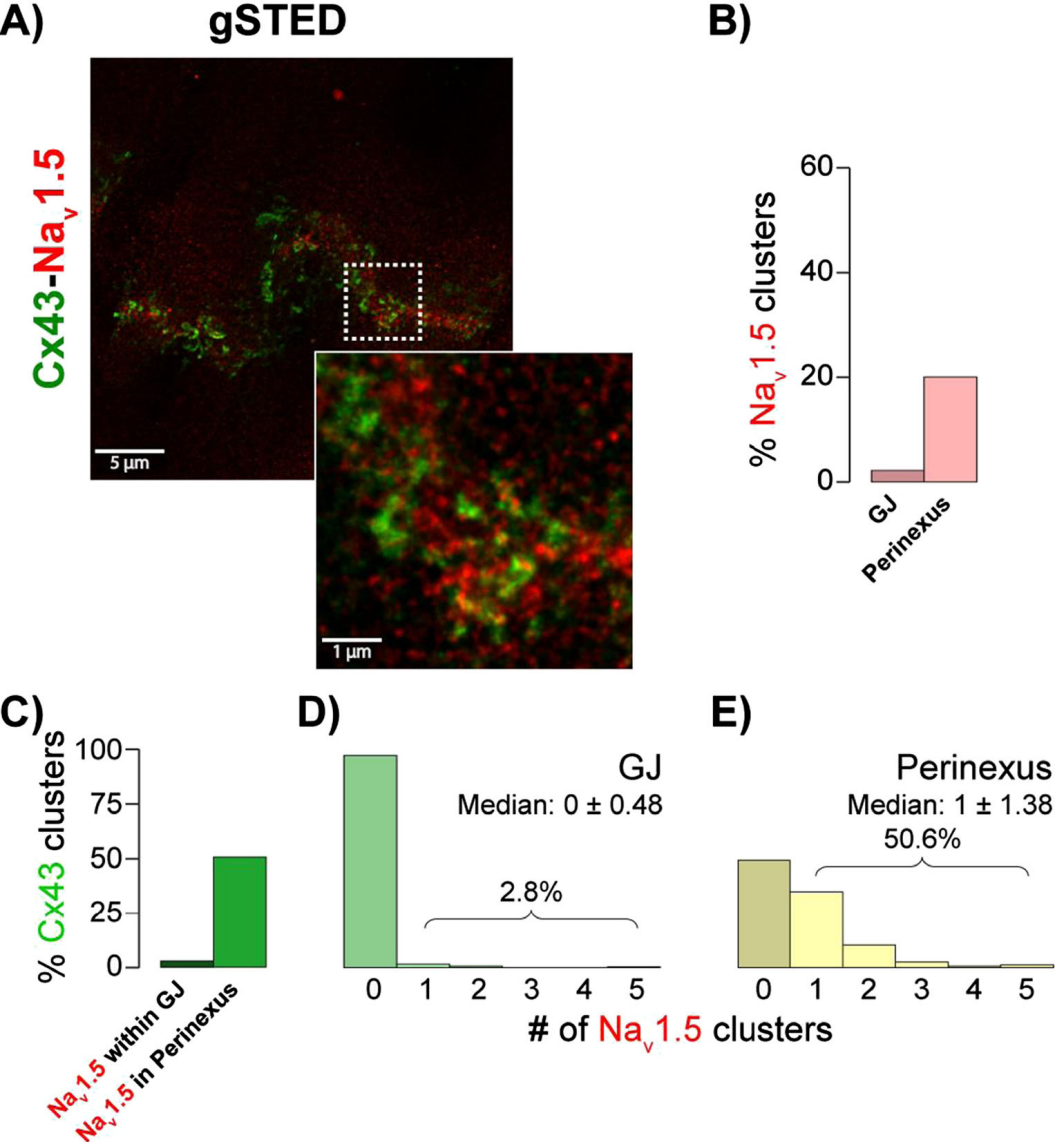
Cx43 and Na_v_1.5 distribution at the ID. **a** Representative gSTED micrograph of guinea pig ventricular sections showing Cx43 (*green*) and Na_v_1.5 (*red*) immunosignals. *Inset* shows high magnification view of the region highlighted by the *dashed white box*. **b** Location of Na_v_1.5 clusters relative to Cx43 clusters (*n* = 3 hearts, 1 tissue section/heart, and 3 images/section). **c** Location of Cx43 clusters relative to Na_v_1.5 clusters and histograms of Cx43 clusters by number of Na_v_1.5 clusters **d** directly overlapping and **e** located within the perinexal region

Next, we assessed the spatial relationship between Na_v_1.5 and Cx43 by confocal and gSTED super-resolution microscopy. Confocal images of guinea pig ventricular sections revealed Na_v_1.5 localization around myocytes, with enrichment at the ID, where signal co-distributed with Cx43 (Fig. 2a–c). Higher magnification views of IDs revealed areas of colocalization but also indicated that Na_v_1.5 and Cx43 were frequently adjacent to each other (Fig. 2d–f). In a representative super-resolution gSTED micrograph (Fig. 3a), punctate Na_v_1.5 staining (red) is present within, but mainly distal or proximal to Cx43 (green) signals. Quantitatively, analysis of the gSTED data yielded a median Cx43 cluster size of 181 ± 19 nm (Supplementary Table), which is below the diffraction-limited resolution of confocal microscopy (∼200 nm) but in the same range as previous estimates obtained by electron microscopy [11]. Further, gSTED imaging suggested that only 2.2 % of Na_v_1.5 punctae directly overlap with Cx43 and 22 % are localized within 200 nm from the edge of Cx43 punctae (Fig. 3b). From a different perspective, gSTED imaging also suggested that 2.8 % of Cx43 clusters had overlapping Na_v_1.5 clusters and 50.6 % had Na_v_1.5 clusters located within the perinexal regions (Fig. 3c). Figure 3d offers a further refinement of this analysis, breaking down Cx43 clusters by the number of overlapping Na_v_1.5 clusters: the vast majority (97.2 %) had none, and the median number of Na_v_1.5 clusters overlapping Cx43 clusters was 0 (standard deviation = 0.48). Figure 3e provides an analysis of Cx43 clusters based on the number of Na_v_1.5 clusters within their perinexi: 34.9 % of Cx43 clusters had one Na_v_1.5 cluster within their perinexal regions and 15.7 % had two or more Na_v_1.5 clusters located within their perinexal regions. In all, 53.4 % of all Cx43 clusters identified had one or more Na_v_1.5 clusters less than 200 nm away, and the median number of Na_v_1.5 clusters located within the perinexus of a given Cx43 cluster was 1 (standard deviation = 1.38). These data suggest that Na_v_1.5 channels closely associating with Cx43 localize mostly adjacent to GJs (i.e., likely within the perinexus), rather than within the GJ plaque proper.

### Conduction dependence on AIE and GJ coupling

We previously published that AIE unmasks conduction slowing in response to small degrees of GJ uncoupling [42]. Consistent with our previous findings, time-control optical mapping experiments revealed that 10 min of AIE alone slowed conduction, preferentially in the transverse direction (CV_T_), and this conduction slowing was maintained for up to 30 min (Supplemental Fig. 1A). Further, AR increased after 10 min of AIE and remained elevated through 30 min of AIE.

**Fig. 4:**
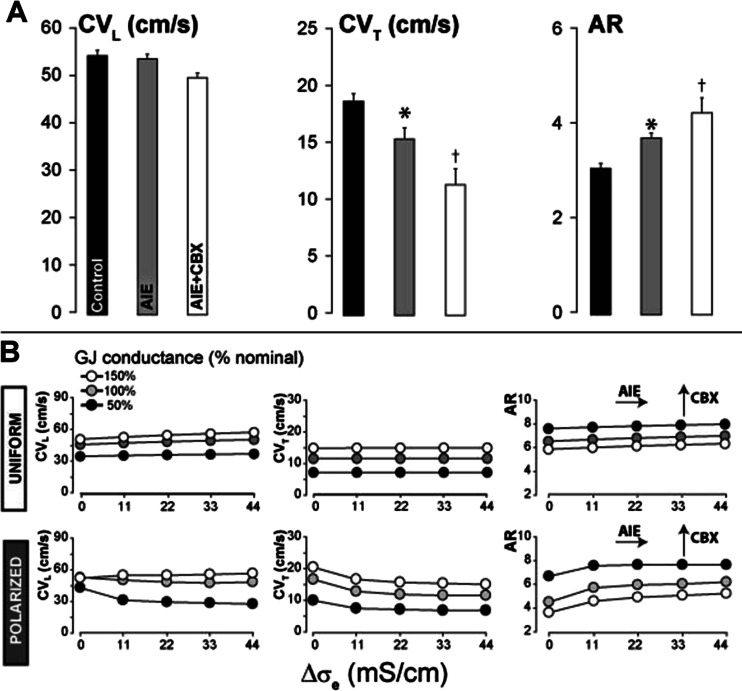
Acute interstitial edema (AIE) slows CV preferentially in the transverse direction of propagation. **a** Experimental results from Veeraraghavan et al., Am J Physiol., 2012 (1). **b** Model predictions of the relationship of CV and AR as a function of σ_e_ and GJ conductance. *Uniform model* (*top*) predicts CV modestly increases and the *polarized model* (*bottom)* predicts CV decreases as σ_e_ increases. Both models predict a rise in AR, but due to changes in CV_L_ in the *uniform* and CV_T_ in the *polarized model*. The *arrows* indicate directional trends caused by AIE and CBX

Pharmacological GJ uncoupling alone with 10 μM CBX slowed conduction within 10 min of perfusion and maintained conduction slowing for 30 min (Supplemental Fig. 1B). The preferential decrease of CV_T_ increased AR as expected. Figure 4a summarizes previously published experimental results demonstrating that AIE or CBX alone slow conduction at 10 min [42]. Independent of the perfusion order, an additional 10 min of both AIE + CBX significantly slowed CV_T_ more than either AIE or CBX alone without significantly reducing longitudinal conduction (CV_L_), thereby increasing AR.

To explore a possible mechanism by which changes in tissue hydration can slow conduction anisotropically, we compared these experimental observations with a previously published mathematical model that explored the relationship between sodium channel distribution and extracellular conductivity on ephaptic coupling [20]. The model was adapted here such that sodium channels were either uniformly distributed around the cell (11 % of channels in ID, *uniform model*) or preferentially localized to the ID (90 % of channels in ID, *polarized model*). Since the model does not incorporate detailed ID ultrastructure, we chose to explore the effects of AIE by increasing both lateral and junctional extracellular conductivities (σ_e_) by 44 mS/cm, a value which produced CV values similar to those observed experimentally.

The *uniform model* in Fig. 4b predicts a modest positive correlation between CV_L_ and σ_e_. However, it does not predict a significant correlation between CV_T_ and σ_e_. The modest increase in AR predicted is therefore mainly due to CV_L_ changes. This is inconsistent with our experimental observations that 1) CV_T_ is more sensitive and negatively correlated to AIE and 2) increased AR is mainly due to CV_T_ changes. The *uniform model* recapitulates experimental findings that GJ uncoupling slows CV, as evidenced by the downward shift in the curves.

The *polarized model*, on the other hand, predicts that CV decreases as σ_e_ increases (Fig. 4b). With GJ uncoupling, this inverse relationship is steepened for CV_L_, and the inverse relationship is mildly flattened for CV_T_. For the explored parameter range, AR increases as σ_e_ increases. GJ uncoupling shifts the AR-σ_e_ relationship upwards, and the finding that AIE + GJ uncoupling produces the largest AR relative to control or AIE conditions alone is supported by this model. These data suggest that AIE can modulate ephaptic coupling particularly during GJ uncoupling and thereby anisotropic conduction.

### Conduction dependence on intermembrane spacing and I_Na_

**Fig. 5:**
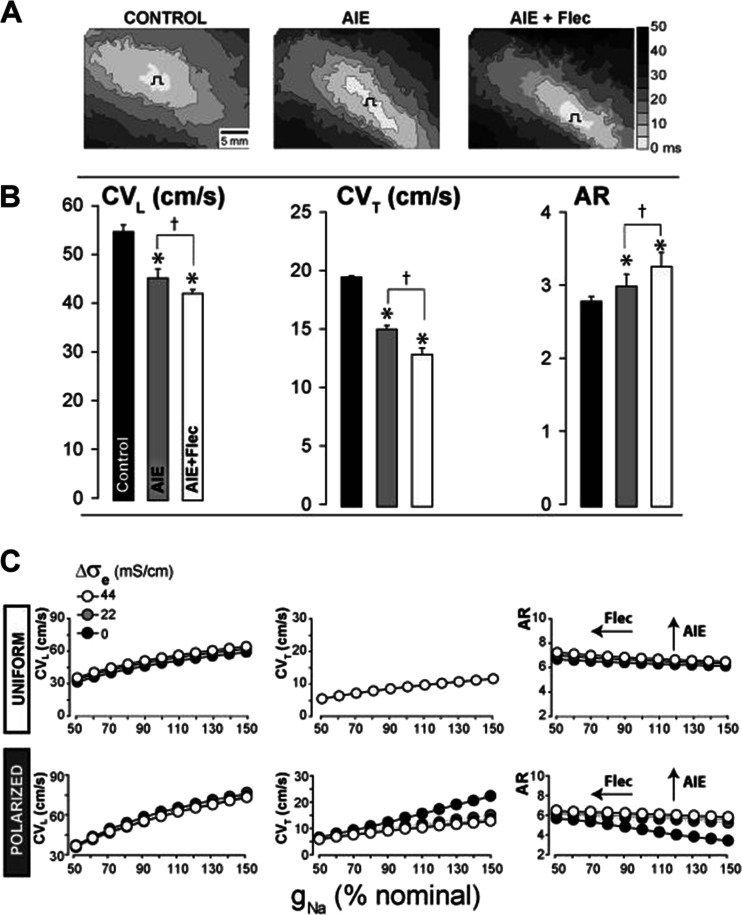
Sodium channel inhibition anisotropically slows conduction during acute interstitial edema (AIE). **a** Representative activation isochrone maps. **b** Longitudinal (*CV*_*L*_) and transverse (*CV*_*T*_) conduction velocity are decreased by AIE and the combination of AIE + Flec (*n* = 5 hearts, *p < 0.05). AIE + Flec slowed CV_L_ and CV_T_ more than AIE alone (†*p* < 0.05). AIE and AIE + Flec increased AR and AIE + Flec increased AR above AIE alone. **c** Model predictions of CV and AR dependence on sodium channel conductance (g_Na_) at different levels of GJ conductance generated using the *uniform model* (*top*) and the *polarized model* (*bottom*). The *arrows* indicate directional trends caused by Flec and AIE

The *polarized model* suggests that dense sodium channel localization at the ID is important to recapitulate our initial and previous results and further suggests that sodium channels may play a novel role in anisotropic conduction. To explore this hypothesis further, we inhibited I_Na_ with 0.5 μM Flec. This slowed conduction uniformly at 10 min and maintained conduction slowing for 30 min (Supplemental Fig. 1C). In a separate set of experiments, AIE once again slowed CV_L_ and CV_T_ at 10 min and increased AR secondary to greater CV_T_ slowing (Fig. 5a, b). During an additional 10 min of AIE + Flec, both CV_L_ and CV_T_ slowed significantly more than during AIE alone. Importantly, in a novel result, AIE + Flec slowed CV_T_ more than AIE alone further increasing AR (Fig. 5b).

We compared these experimental results to the *uniform* and *polarized models* to determine whether ephaptic coupling could explain the increased sensitivity of CV_T_ to sodium channel inhibition during AIE. In Fig. 5c, σ_e_ is now represented by the family of curves, and CV and AR are plotted as a function of g_Na_. Raising σ_e_ increased CV_L_ in the *uniform model* as evidenced by the modest upward shift of the curves, while CV_T_ remained unaltered. Thus, the *uniform model* predicted that reducing g_Na_, as would occur with Flec, would only modestly affect AR even during AIE.

Increasing σ_e_ in the *polarized model* decreased both CV_L_ and CV_T_ as evidenced by the modest downward shift in the curves (Fig. 5c). The *polarized model* further predicts an upward shift in the relationship between AR and g_Na_ stemming from the greater sensitivity of CV_T_ to σ_e_ changes. The finding from model predictions that AR is less sensitive to g_Na_ during edema is inconsistent with our data, but the prediction that the largest ARs are observed during conditions of reduced g_Na_, and AIE is consistent with experimental findings in Fig. 5b.

### Conduction dependence on GJ coupling and I_Na_

**Fig. 6:**
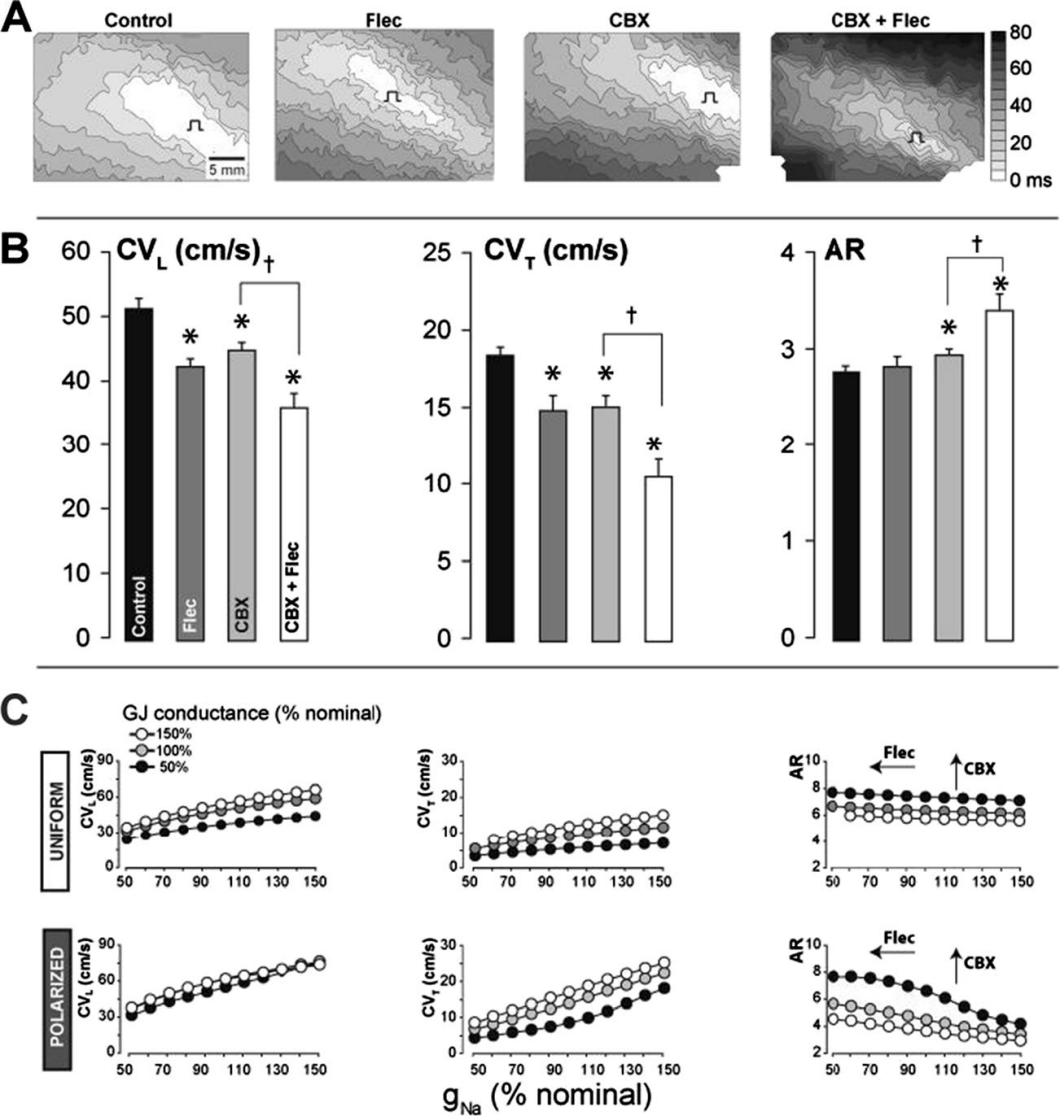
Sodium channel inhibition anisotropically slows conduction during GJ inhibition. **a** Representative activation isochrone maps. **b** Longitudinal (*CV*_*L*_) and transverse (*CV*_*T*_) conduction velocity are decreased by Flec, CBX, and combination of CBX + Flec (*n* = 5 hearts, *p < 0.05). CBX + Flec slowed CV_L_ and CV_T_ more than CBX alone (†*p* < 0.05). CBX and CBX + Flec increased AR and CBX + Flec increased AR above CBX alone. **c** Model predictions of conduction velocity and AR as a function of sodium channel conductance (g_Na_) at different levels of GJ conductance generated using the *uniform model* (*top*) and *polarized model* (*bottom*). The *arrows* indicate directional trends caused by Flec and CBX

In order to probe the relative roles of GJ and ephaptic coupling, we next investigated conduction dependence on I_Na_ during normal and compromised GJ coupling. Again, 10 min of Flec perfusion reduced CV_L_ and CV_T_ without altering AR (Fig. 6a, b). Ten minutes of CBX perfusion alone reduced CV_L_ and CV_T_ and increased AR (Fig. 6a, b), and these effects were maintained through 30 min of perfusion (Supplemental Fig. 1C). AR increased due to increased sensitivity of CV_T_ to CBX. Importantly, 10 additional minutes of both CBX + Flec further decreased CV_L_ and CV_T_ more than either intervention alone regardless of perfusion order, but it preferentially decreased CV_T_ and increased AR (Fig. 6a, b). In short, Flec alone had no effect on AR. Yet, during GJ uncoupling, I_Na_ inhibition with Flec preferentially slowed CV_T_ and increased AR.

In comparison with these experimental results, the *uniform model* predicted a rise in CV_L_ and CV_T_ with a modest decrease in AR as g_Na_ was increased (Fig. 6c, top panels). Decreasing GJ coupling decreased CV for all values of g_Na_, increasing AR in a relatively g_Na_-independent manner.

In the *polarized model*, increasing g_Na_ increased CV_T_ to a greater extent than CV_L_, which subsequently decreased AR (Fig. 6c, bottom panels). Decreasing GJ coupling decreased CV for all values of g_Na_ and resulted in a steeper relationship between AR and g_Na_ than predicted by the *uniform model*. Thus, importantly, the *polarized model*’*s* predictions are consistent with experimentally observed increased AR secondary to preferential CV_T_ slowing during GJ uncoupling and sodium channel inhibition.

### Arrhythmia incidence

**Fig. 7:**
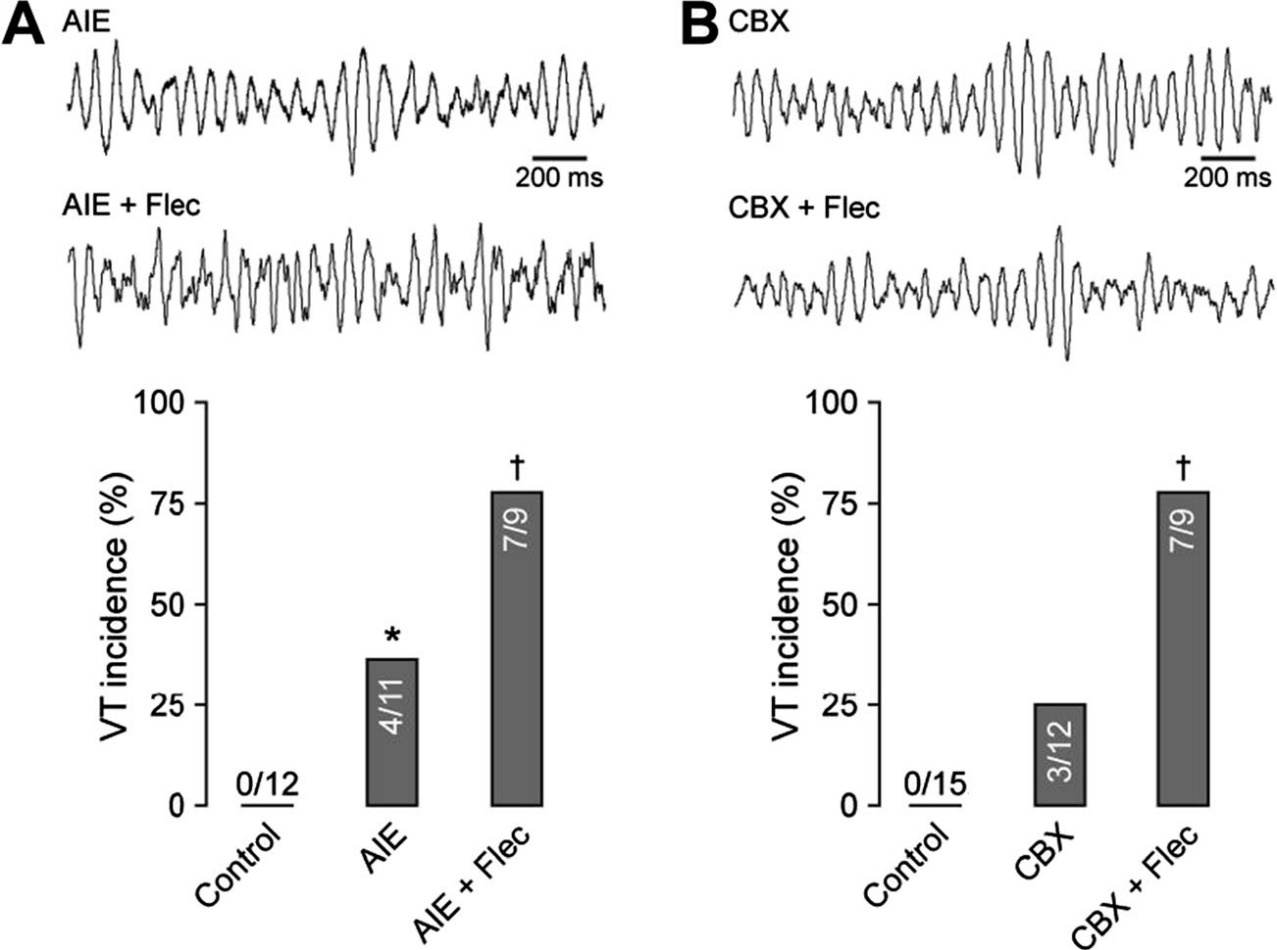
Sodium channel inhibition increases incidence of spontaneous ventricular arrhythmias above GJ uncoupling or AIE alone. Representative ECG traces and incidence of spontaneous VTs during **a** GJ uncoupling or **b** AIE. *p < 0.05 vs. control; †*p* < 0.05 vs. AIE

Due to the high correlation between conduction slowing, particularly anisotropic conduction slowing [17], and arrhythmia susceptibility, we quantified the incidence of spontaneous VTs during all experimental conditions which is summarized in Fig. 7 along with representative ECG traces of VTs. CBX + Flec resulted in a significantly higher VT incidence relative to CBX alone (Fig. 7a). Similarly, while AIE by itself was proarrhythmic relative to control, AIE + Flec inhibition increased VT incidence even further (Fig. 7b). In short, conditions which slowed conduction anisotropically were also associated with the highest arrhythmia burden.

## Discussion

We present here a combination of experimental evidence ranging from structural data at the nano-scale to functional data at the whole organ scale as well as computer modeling to suggest a role for ephaptic coupling in cardiac conduction. Previous in silico studies suggest that the structural requirements for ephaptic coupling include: 1) close apposition of adjacent cell membranes and 2) a high density of depolarizing ion channels [18, 24, 36]. Here, we characterize one such structure—the perinexus—possessing both these characteristics and offer novel evidence that this structure can be dynamically regulated. We further provide functional evidence for conduction phenomena that are well explained by ephaptic coupling but cannot be accounted for by GJ coupling alone—as assessed by mathematical models.

## Structural evidence for a cardiac ephapse

### ID ultrastructure

The perinexus, defined as a peri-GJ microdomain containing undocked Cx43 hemichannels [28, 29], was identified in the present study by electron micrographs where immunogold labeled Cx43 hemichannels were observed within non-junctional perinexal membranes. We demonstrate for the first time that the intermembrane spacing within the perinexus is nominally less than 10 nm, a range theoretically predicted to support ephaptic coupling [18, 20, 24]. Further, we provide the first evidence that intermembrane spacing within the perinexus was significantly increased (to ∼18 nm) during mannitol-induced AIE, a condition we previously demonstrated increases gross interstitial volume and slows conduction [42]. While intermembrane distances at sites further than 200 nm from the edge of the GJ plaque were not significantly altered by AIE, this does not preclude other ID microdomains being affected by AIE or functioning as a cardiac ephapse.

### Sodium channel localization

The second theoretical requirement for an ephapse is a high density of depolarizing ion channels within microdomains of close membrane apposition. In micrographs of guinea pig ventricular sections, we observed enrichment of both Na_v_1.5 and Cx43 at the ID, as previously demonstrated by us and others [23, 26, 29]. Further, using gSTED super-resolution microscopy, we provide quantitative evidence that 51 % of Cx43 clusters had Na_v_1.5 clusters located less than 200 nm from their edges, with these regions accounting for 22 % of Na_v_1.5 clusters identified. This distance corresponds to the extent of the perinexus as measured by TEM in this study and previous in situ co-immunoprecipitation (Duolink) experiments [29]. These data mark the first identification of a sodium channel-rich ID microdomain where intermembrane spacing between adjacent myocytes can be dynamically modulated. As such, structural evidence is provided that the perinexus may function as a cardiac ephapse.

Additionally, we observed considerable Na_v_1.5 signal located beyond 200 nm from the GJ edge and recent evidence identified Na_v_1.5 enrichment adjacent to desmosomes [1]. Taken in this context, the structural characteristics of the perinexus may constitute a blueprint with which to identify other sodium channel-rich ID microdomains that could support ephaptic coupling. In other words, other Na_v_1.5-rich ID microdomains may exist, where intermembrane spacing may be close enough to support ephaptic coupling. Likewise, ion concentrations within such ID microdomains could be modulated by other ID-localized channel types, provided that they are active during the action potential upstroke. Indeed, Cx43 hemichannels have been suggested to play a key role in ephaptic coupling in the vertebrate retina [43]; however, in the heart, hemichannels are thought to remain closed during normal physiological conditions and to open only during ischemia [31, 34, 44]. Future studies using specific Cx43 hemichannel blockers [15] will be useful in determining whether hemichannels play a role in normal physiology.

## Functional evidence for ephaptic coupling

### Intermembrane spacing and conduction

Consistent with our previous results [42], we determined that, in the ventricular myocardium, AIE preferentially decreased CV_T_, increased AR, and precipitated arrhythmias. Our data stand in apparent contrast to those of Fleischauer et al. who reported that increasing the concentration of the large molecular weight colloid, dextran (70,000 Da), decreased fiber diameter, and slowed conduction (longitudinally) in papillary muscles [8]. We, on the other hand, demonstrated in this and a previous study that increasing the concentration of the small molecular weight crystalloid mannitol (MW 182.1 Da) increased interstitial volume and slowed conduction, particularly in the transverse direction [42]. One possible explanation for the apparent differences between the two studies is that fiber diameter changes could stem from changes in vascular, interstitial and/or intracellular volumes, thus masking changes in one or more compartments. We now provide additional evidence that mannitol not only increases gross edema, but also increases perinexal separation and this phenomenon correlates with slowed ventricular conduction.

In the light of previous in silico studies, the observed association between increased perinexal width and conduction slowing is suggestive of ephaptic coupling [18, 19, 24]. Utilizing a computational model of conduction longitudinal and transverse to fibers, we demonstrate that the model with *uniform s*odium channel cellular distribution predicted a modest increase in CV during AIE. However, our *polarized model* with Na_v_1.5 enrichment at the ID predicted conduction slowing during AIE, particularly along the transverse direction. While the CV_L_ slowing predicted by our model, as well as that which we experimentally measured, agree with single strand model predictions of longitudinal conduction during sodium channel polarization [18, 24]; this is the first experimentally supported computational demonstration of greater transverse conduction sensitivity to modulating ephaptic coupling. It should be noted that 90 % of sodium channels were localized to the ID in the *polarized model* since this distribution yielded trends consistent with experimental data. While this value is not directly drawn from experimental measurements, it is consistent with a previous report that peak sodium current at the ends of myocytes was 7.5 times as large as that occurring on lateral membranes and that lateral Na_v_1.5 channels may be largely inactive at resting potentials [22].

### Sodium channels and conduction

Further support for the hypothesis that ephaptic coupling is mediated by dense sodium channel localization to the ends of myocytes comes from the novel finding presented here that I_Na_ inhibition during AIE slowed conduction anisotropically. Our data demonstrating that Flec slows conduction in a direction-independent manner under control conditions is entirely consistent with previous studies using similar doses of Flec, and it is generally accepted that I_Na_, as a key determinant of excitability, modulates conduction in a direction-independent manner [17]. Yet, it is demonstrated herein that I_Na_ inhibition during AIE preferentially slowed transverse conduction, increased anisotropy, and increased arrhythmia incidence relative to AIE.

Once again, the *uniform model* diverged from experimental observations. The predictions of the *polarized model* on the other hand matched experimental observations that AIE + Flec preferentially slowed CV_T_ and significantly increased AR. Consequently, sodium channel inhibition can anisotropically slow conduction under conditions such as AIE where ephaptic coupling may be compromised.

### Gap junctions, sodium channels, and conduction

We next investigated anisotropic conduction dependence on I_Na_ during GJ uncoupling. Whereas I_Na_ inhibition by itself slowed conduction isotropically, pharmacological GJ uncoupling preferentially slowed transverse conduction and increased AR, consistent with previous studies [30, 42]. In the present study, we provide novel experimental and computational modeling data that partial I_Na_ blockade during GJ uncoupling further slows conduction in a direction-dependent manner. These results echo those of Stein et al. who demonstrated preferential transverse over longitudinal conduction slowing in the ventricle of Cx43/Na_v_1.5 double heterozygous knockout mice relative to Cx43 heterozygous null mice [38]. While the observations in Na_v_1.5 knockout mice could reflect confounding changes in other proteins such as Cx43 [16], our results demonstrate that I_Na_ inhibition can anisotropically slow conduction during GJ uncoupling. Stein et al. also found greater arrhythmia susceptibility when both Cx43 and Na_v_1.5 gene dosage were reduced by 50 %, relative to a 50 % reduction in either protein alone. This concurs with our observation of greater spontaneous VT incidence during combined pharmacologic reduction of I_Na_ and GJ coupling relative to GJ uncoupling alone.

Simulations further support the role of I_Na_ in modulating anisotropic conduction. The *uniform model* predicts isotropic conduction slowing, consistent with this and other experimental results [4, 41] and simulations without subcellular sodium channel distribution [17, 33]. However, this model could not predict anisotropic conduction slowing due to combined I_Na_ and GJ inhibition. Importantly, the *polarized model* supports the experimental data that I_Na_ inhibition during GJ uncoupling heterogeneously slows CV_L_ and CV_T_ leading to elevated AR.

### Limitations

Our data should be interpreted with care as although the *polarized model* well fits our experimental data, it may not incorporate all key structural details of IDs. For instance, the ID is a complex 3-dimensional structure with a surface area significantly larger than the cross-sectional area of a myocyte [9, 12, 14]. Additionally, the model was tuned to match experimental trends in conduction rather than absolute values. While there are clearly many interesting areas still to explore with respect to sodium channel distribution and modes of electrical coupling, these unknowns do not detract from the central finding that inhibiting sodium channels anisotropically slows conduction. Further, the data indicate that modulation of the closeness of apposition of cell membranes with enriched densities of sodium channels, such as those occurring at the perinexus, could have important effects on anisotropic conduction.

## Conclusion

We present evidence for a candidate structure for the cardiac ephapse with close membrane apposition and a high density of Na_v_1.5 that can be modulated in a whole heart preparation. Importantly, we provide experimental evidence that anisotropic cardiac conduction is dependent on gap junctional as well as sodium channel localization at the intercalated disk by a mechanism consistent with the computational modeling predictions of ephaptic coupling. There is now increasing evidence suggesting that to better understand the arrhythmic effects of gap junction remodeling, sodium channel modulation, and changes in spacing between myocytes, future studies of cardiac conduction must be attentive to the details of cardiac tissue ultrastructure.

## Electronic supplementary material

Below is the link to the electronic supplementary material.

ESM 1 (DOC 719 kb)
