# Supplementary material for: Sodium channels in the Cx43 gap junction perinexus may constitute a cardiac ephapse: an experimental and modeling study
Source: Pflugers Arch. 2015 Jan 13;467(10):2093–105. doi: 10.1007/s00424-014-1675-z (PMC4500747; doi:10.1007/s00424-014-1675-z)
Supplement: Supplementary file 1 — (DOC 719 kb) [file 424_2014_1675_MOESM1_ESM.doc]

**Supplement**

**Supplemental Table**

| **Cx43 - Na_v_1.5 gSTED - Quantification** | |
| --- | --- |
| Median distance from Cx43 clusters to nearest Na_v_1.5 cluster | 51 ± 31 nm |
| Correlation between Cx43 cluster size and number of Na_v_1.5 clusters within perinexus | R^2^ = 0.39 |
| Median equivalent diameter of Cx43 clusters | 181 ± 19 nm |
| Median equivalent diameter of Na_v_1.5 clusters | 161 ± 4 nm |

**
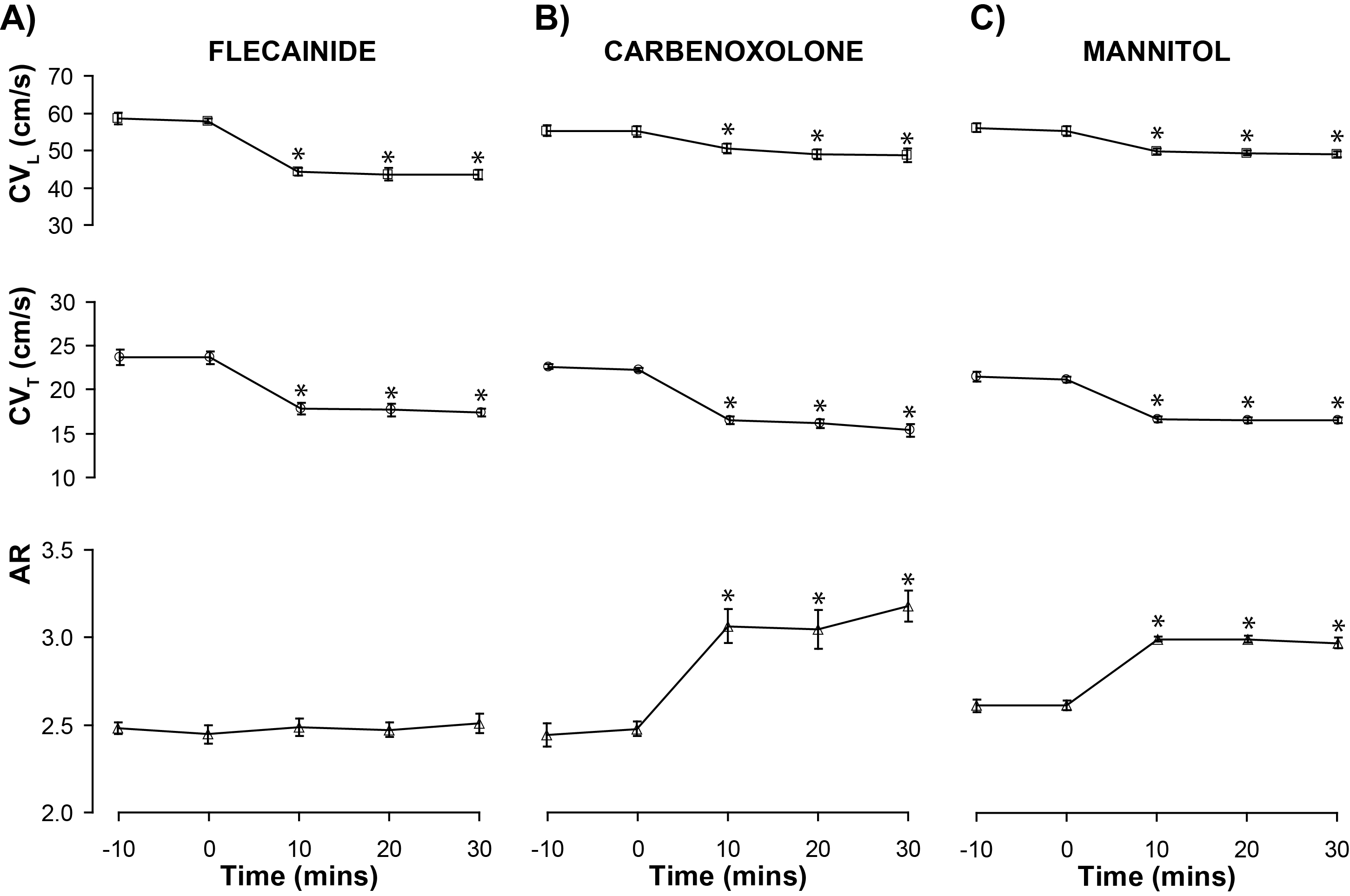
**

**Supplemental Figure 1. Time control experiments demonstrating conduction changes during perfusion of A)** flecainide**, B)** carbenoxolone and **C)** mannitol**.** In all cases, CV_L_ and CV_T_ were decreased after 10 minutes of perfusion and remained stable through 30 minutes of perfusion. Flecainide did not alter AR whereas both carbenoxolone and mannitol increased it relative to control (t = 0 minutes). * p <0.05 vs. time 0.
